# DEVELOPMENT AND TESTING AN INTERPROFESSIONAL PLIÉ TRAINING PROGRAM FOR VA NURSING HOME STAFF

**DOI:** 10.1093/geroni/igac059.681

**Published:** 2022-12-20

**Authors:** Jennifer Lee, Francesca Nicosia, Catherine Pham, Nikita Shirsat, Matthew Miller, Margaret Chesney, Deborah Barnes

**Affiliations:** San Francisco VA Health Care System / Together Senior Health, San Francisco, California, United States; University of California, San Francisco, San Francisco, California, United States; San Francisco VA Health Care System, San Francisco, California, United States; UC Irvine School of Medicine, Irvine, California, United States; University of California, San Francisco, San Francisco, California, United States; University of California, San Francisco, San Francisco, California, United States; University of California, San Francisco, San Francisco, California, United States

## Abstract

In this study, we collected formative data from key stakeholders (nursing home staff and leadership, dementia care experts), developed, pilot tested, and refined a 10-week remote training program at 2 VA nursing homes with 15 interprofessional staff (e.g., recreation therapists, social workers, registered nurses). Training components included weekly livestreaming didactic sessions for staff that demonstrated PLIÉ guiding principles and themes. Weekly experiential sessions, facilitated by the lead PLIÉ instructor, included staff and residents with dementia participating together online. All training sessions were video recorded and available for review along with a training manual. Staff completed weekly surveys, post-training focus groups, and expressed high confidence using 6 of 8 PLIÉ principles; 100% rated their overall training satisfaction as “very good” or “excellent.” The remote PLIÉ training was an efficient model for staff training that leveraged interprofessional knowledge and skills. This presentation includes an experiential demonstration of PLIÉ in action.

